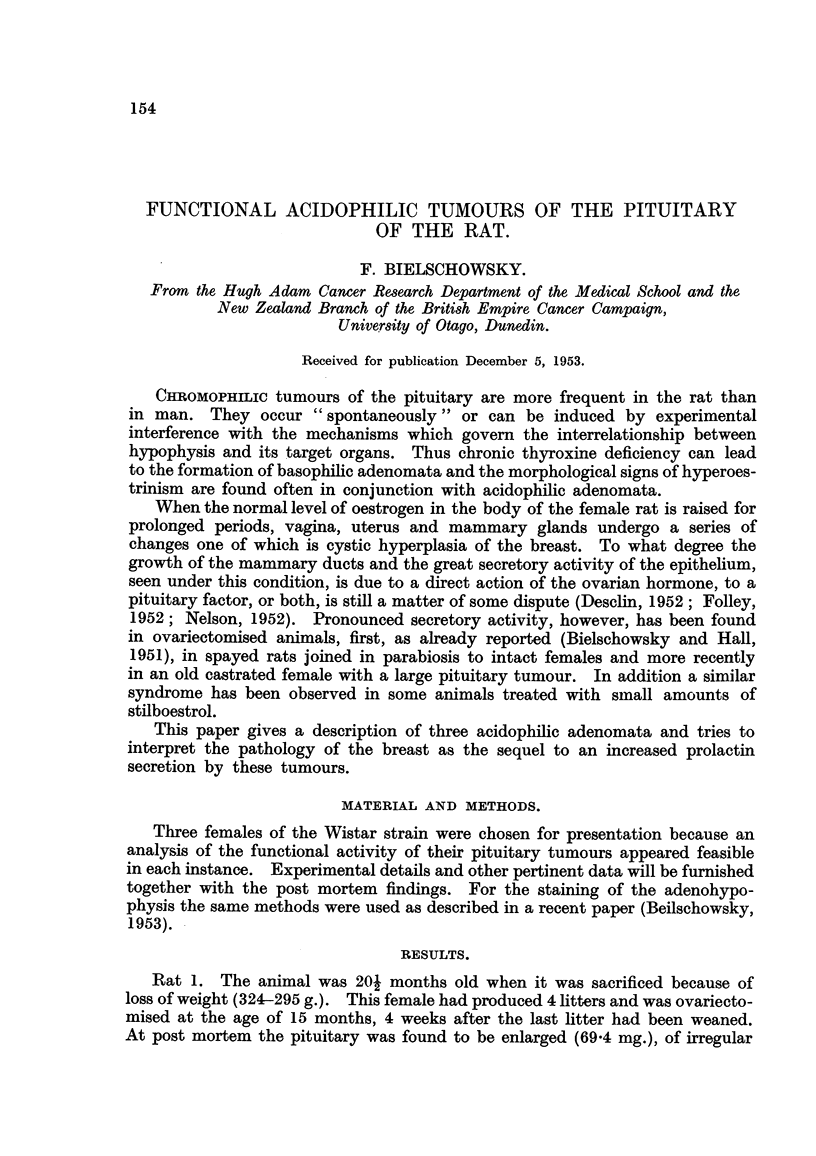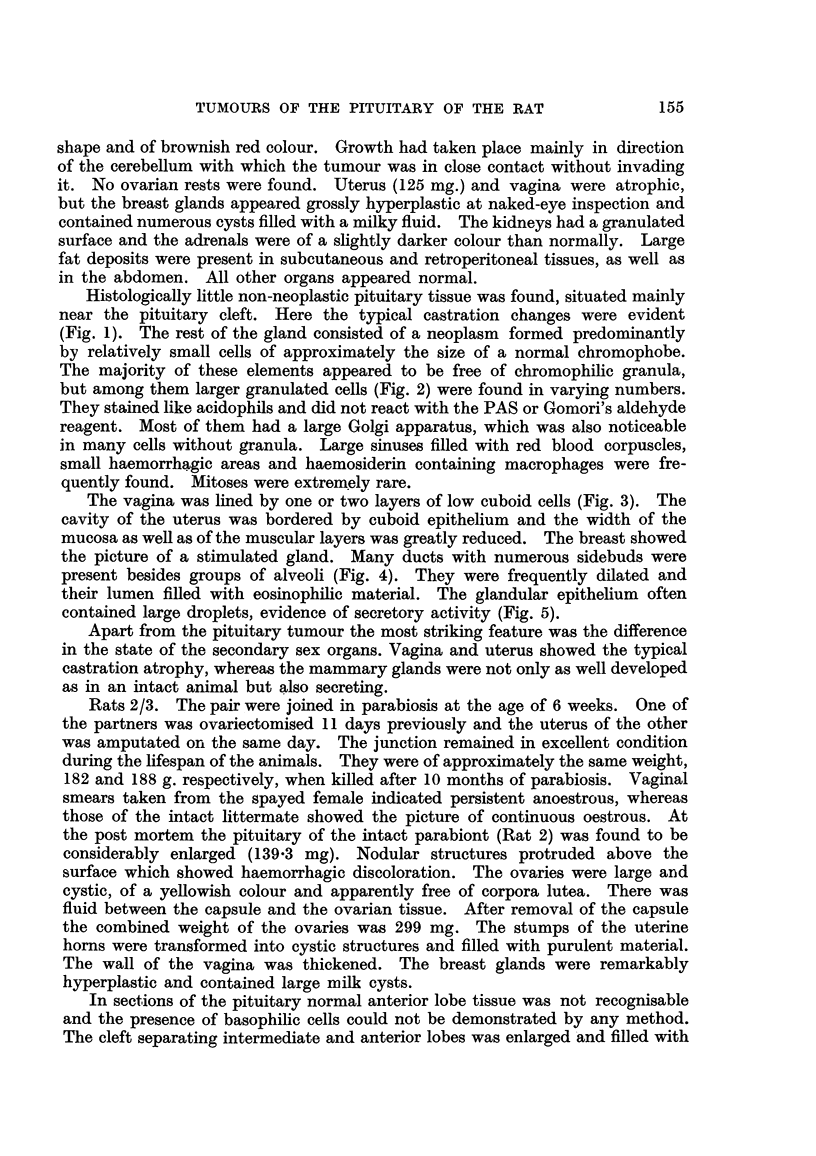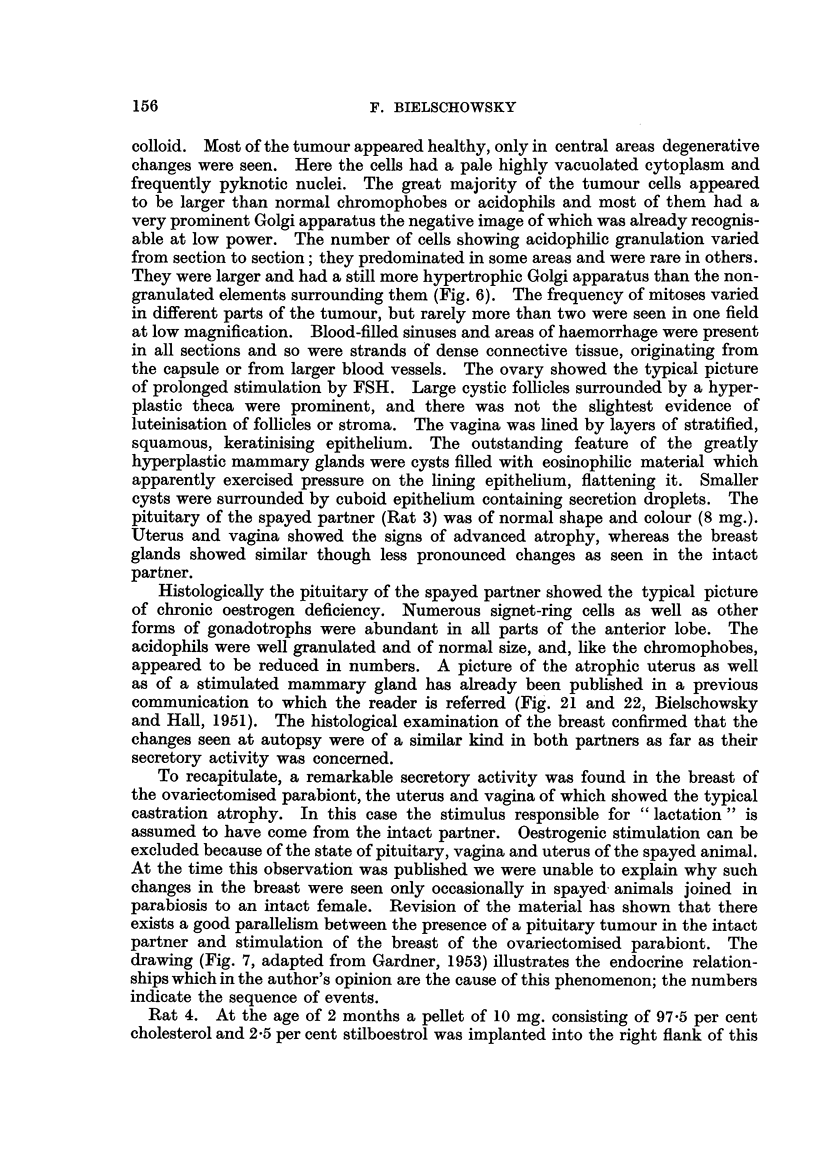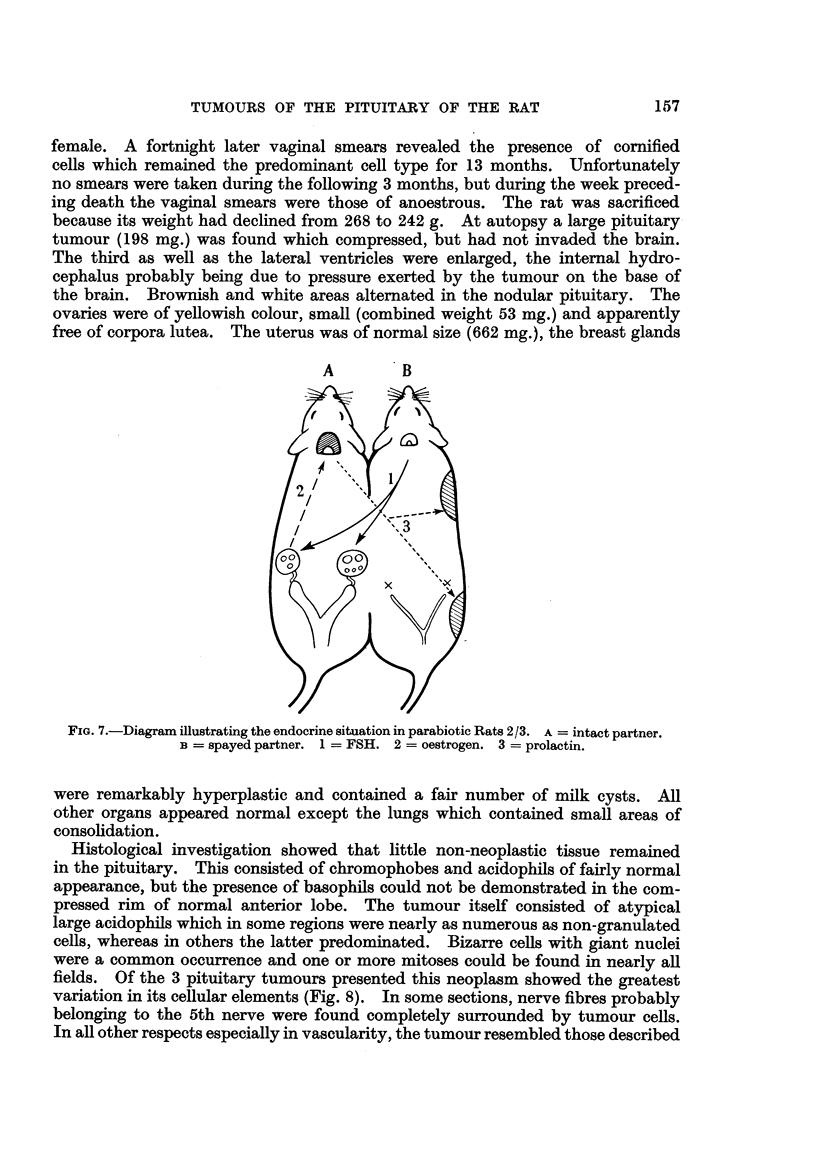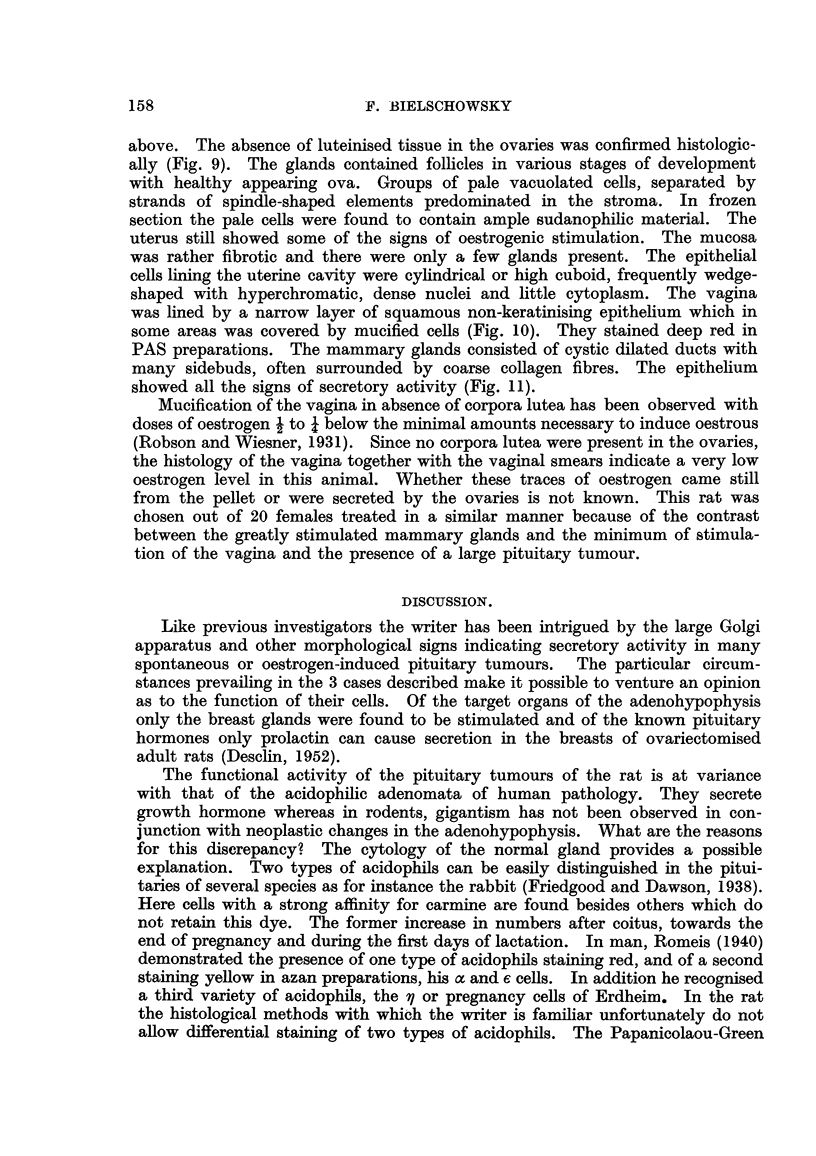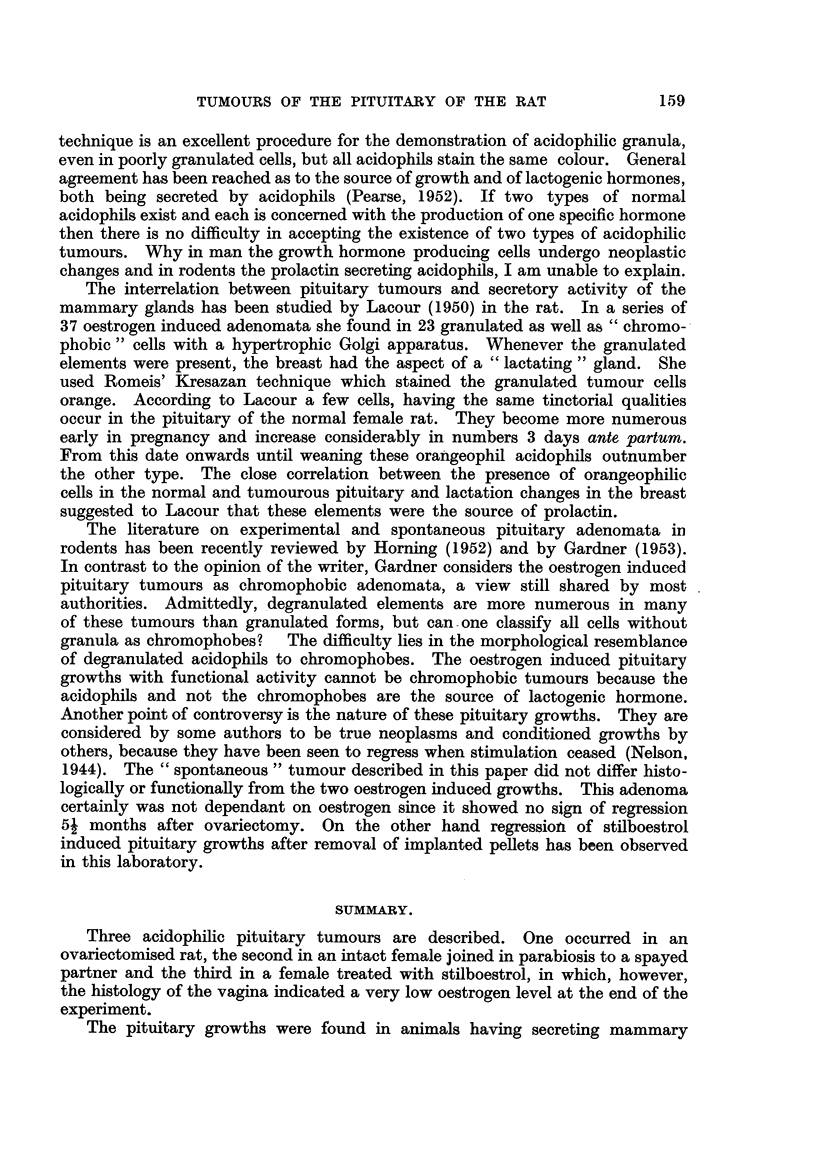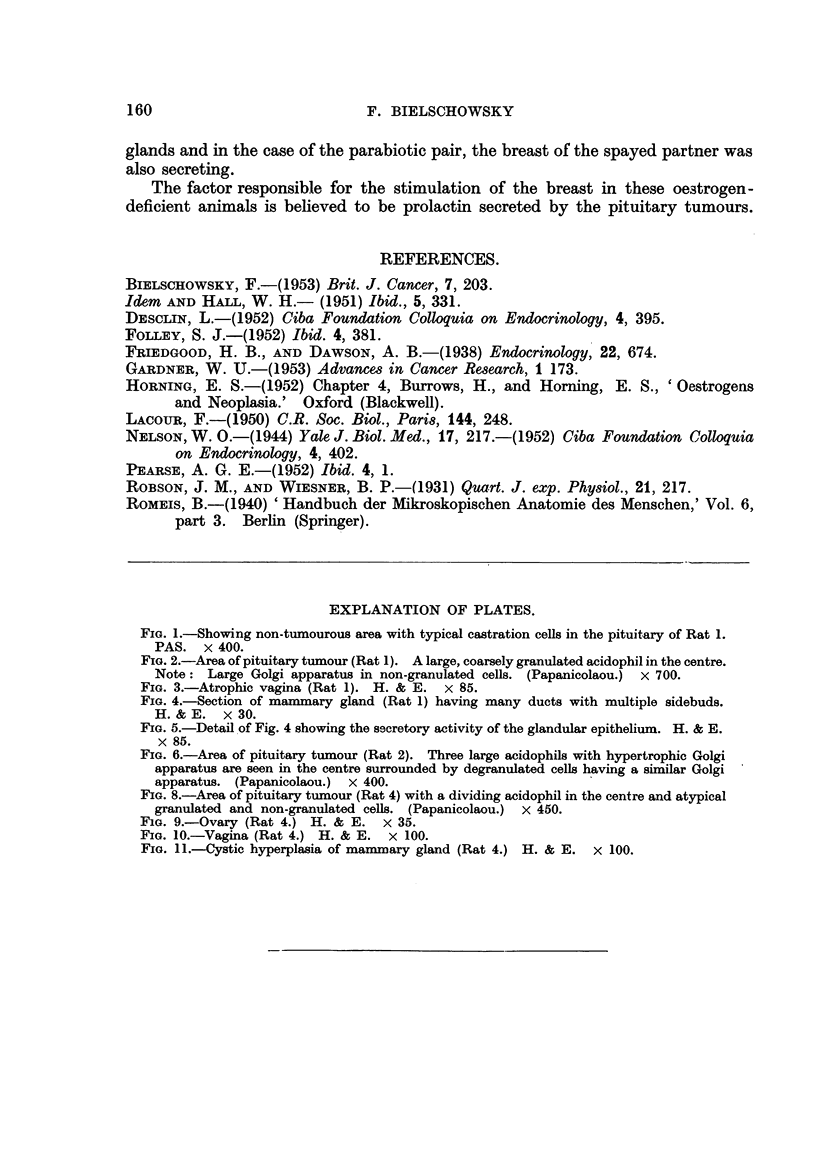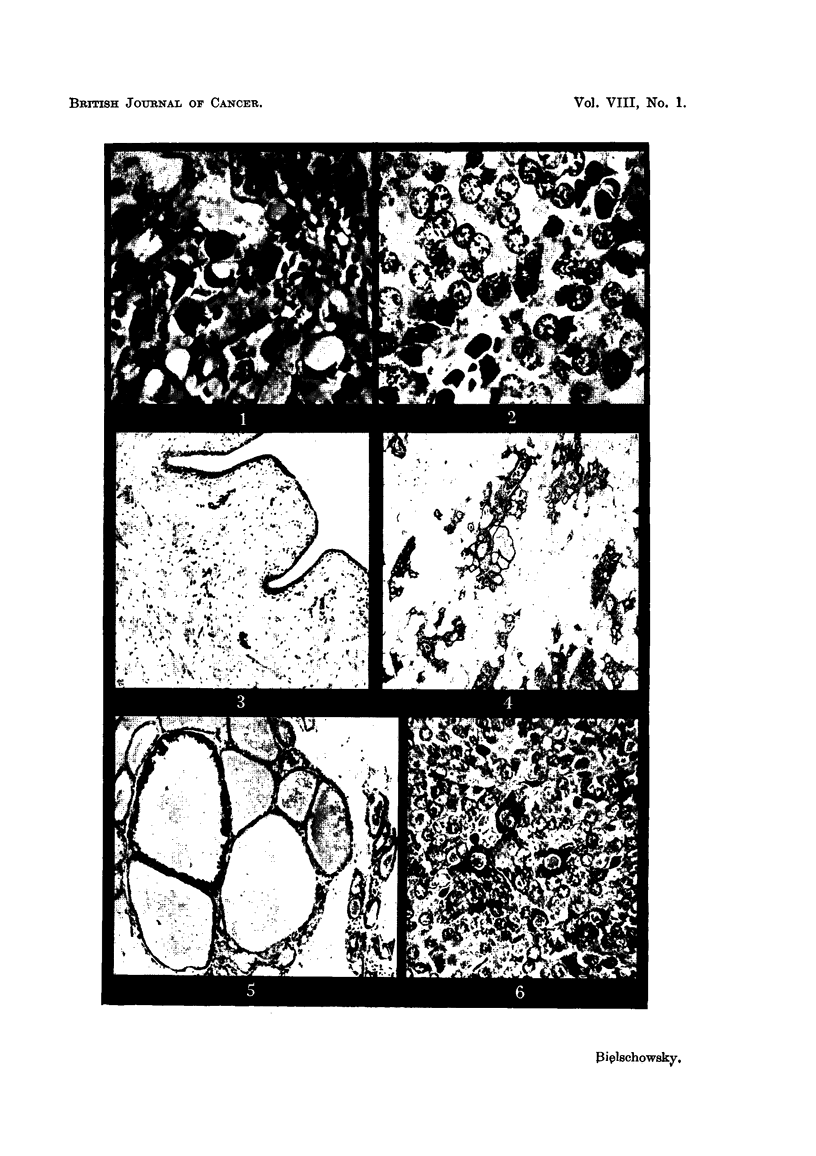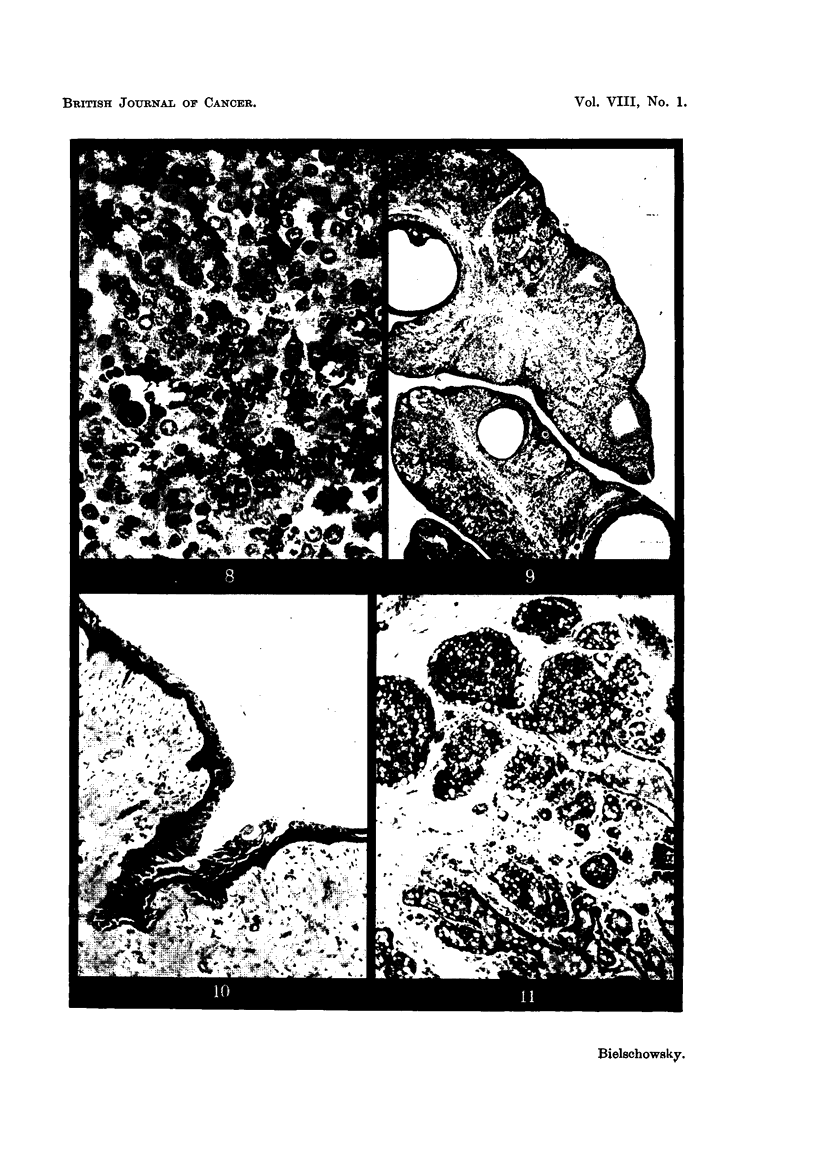# Functional Acidophilic Tumours of the Pituitary of the Rat

**DOI:** 10.1038/bjc.1954.13

**Published:** 1954-03

**Authors:** F. Bielschowsky

## Abstract

**Images:**


### FUNCTIONAL ACIDOPHILIC TUMOURS OF THE PITUITARY OF THE RAT.

F. BIELSCHOWSKY.

*From the Hugh Adam Cancer Research Department of the Medical School and the New Zealand Branch of the British Empire Cancer Campaign, University of Otago, Dunedin.*

Received for publication December 5, 1953.

CHROMOPHILIC tumours of the pituitary are more frequent in the rat than in man. They occur "spontaneously" or can be induced by experimental interference with the mechanisms which govern the interrelationship between hypophysis and its target organs. Thus chronic thyroxine deficiency can lead to the formation of basophilic adenomata and the morphological signs of hyperoestrinism are found often in conjunction with acidophilic adenomata.

When the normal level of oestrogen in the body of the female rat is raised for prolonged periods, vagina, uterus and mammary glands undergo a series of changes one of which is cystic hyperplasia of the breast. To what degree the growth of the mammary ducts and the great secretory activity of the epithelium, seen under this condition, is due to a direct action of the ovarian hormone, to a pituitary factor, or both, is still a matter of some dispute (Desclin, 1952; Folley, 1952; Nelson, 1952). Pronounced secretory activity, however, has been found in ovariectomised animals, first, as already reported (Bielschowsky and Hall, 1951), in spayed rats joined in parabiosis to intact females and more recently in an old castrated female with a large pituitary tumour. In addition a similar syndrome has been observed in some animals treated with small amounts of stilboestrol.

This paper gives a description of three acidophilic adenomata and tries to interpret the pathology of the breast as the sequel to an increased prolactin secretion by these tumours.

### MATERIAL AND METHODS.

Three females of the Wistar strain were chosen for presentation because an analysis of the functional activity of their pituitary tumours appeared feasible in each instance. Experimental details and other pertinent data will be furnished together with the post mortem findings. For the staining of the adenohypophysis the same methods were used as described in a recent paper (Beilschowsky, 1953).

### RESULTS.

Rat 1. The animal was 20½ months old when it was sacrificed because of loss of weight (324–295 g.). This female had produced 4 litters and was ovariectomised at the age of 15 months, 4 weeks after the last litter had been weaned. At post mortem the pituitary was found to be enlarged (69·4 mg.), of irregular shape and of brownish red colour. Growth had taken place mainly in direction of the cerebellum with which the tumour was in close contact without invading it. No ovarian rests were found. Uterus (125 mg.) and vagina were atrophic, but the breast glands appeared grossly hyperplastic at naked-eye inspection and contained numerous cysts filled with a milky fluid. The kidneys had a granulated surface and the adrenals were of a slightly darker colour than normally. Large fat deposits were present in subcutaneous and retroperitoneal tissues, as well as in the abdomen. All other organs appeared normal.

Histologically little non-neoplastic pituitary tissue was found, situated mainly near the pituitary cleft. Here the typical castration changes were evident (Fig. 1). The rest of the gland consisted of a neoplasm formed predominantly by relatively small cells of approximately the size of a normal chromophobe. The majority of these elements appeared to be free of chromophilic granula, but among them larger granulated cells (Fig. 2) were found in varying numbers. They stained like acidophils and did not react with the PAS or Gomori's aldehyde reagent. Most of them had a large Golgi apparatus, which was also noticeable in many cells without granula. Large sinuses filled with red blood corpuscles, small haemorrhagic areas and haemosiderin containing macrophages were frequently found. Mitoses were extremely rare.

The vagina was lined by one or two layers of low cuboid cells (Fig. 3). The cavity of the uterus was bordered by cuboid epithelium and the width of the mucosa as well as of the muscular layers was greatly reduced. The breast showed the picture of a stimulated gland. Many ducts with numerous sidebuds were present besides groups of alveoli (Fig. 4). They were frequently dilated and their lumen filled with eosinophilic material. The glandular epithelium often contained large droplets, evidence of secretory activity (Fig. 5).

Apart from the pituitary tumour the most striking feature was the difference in the state of the secondary sex organs. Vagina and uterus showed the typical castration atrophy, whereas the mammary glands were not only as well developed as in an intact animal but also secreting.

Rats 2/3. The pair were joined in parabiosis at the age of 6 weeks. One of the partners was ovariectomised 11 days previously and the uterus of the other was amputated on the same day. The junction remained in excellent condition during the lifespan of the animals. They were of approximately the same weight, 182 and 188 g. respectively, when killed after 10 months of parabiosis. Vaginal smears taken from the spayed female indicated persistent anoestrous, whereas those of the intact littermate showed the picture of continuous oestrous. At the post mortem the pituitary of the intact parabiont (Rat 2) was found to be considerably enlarged (139·3 mg). Nodular structures protruded above the surface which showed haemorrhagic discoloration. The ovaries were large and cystic, of a yellowish colour and apparently free of corpora lutea. There was fluid between the capsule and the ovarian tissue. After removal of the capsule the combined weight of the ovaries was 299 mg. The stumps of the uterine horns were transformed into cystic structures and filled with purulent material. The wall of the vagina was thickened. The breast glands were remarkably hyperplastic and contained large milk cysts.

In sections of the pituitary normal anterior lobe tissue was not recognisable and the presence of basophilic cells could not be demonstrated by any method. The cleft separating intermediate and anterior lobes was enlarged and filled with colloid. Most of the tumour appeared healthy, only in central areas degenerative changes were seen. Here the cells had a pale highly vacuolated cytoplasm and frequently pyknotic nuclei. The great majority of the tumour cells appeared to be larger than normal chromophobes or acidophils and most of them had a very prominent Golgi apparatus the negative image of which was already recognisable at low power. The number of cells showing acidophilic granulation varied from section to section; they predominated in some areas and were rare in others. They were larger and had a still more hypertrophic Golgi apparatus than the non-granulated elements surrounding them (Fig. 6). The frequency of mitoses varied in different parts of the tumour, but rarely more than two were seen in one field at low magnification. Blood-filled sinuses and areas of haemorrhage were present in all sections and so were strands of dense connective tissue, originating from the capsule or from larger blood vessels. The ovary showed the typical picture of prolonged stimulation by FSH. Large cystic follicles surrounded by a hyperplastic theca were prominent, and there was not the slightest evidence of luteinisation of follicles or stroma. The vagina was lined by layers of stratified, squamous, keratinising epithelium. The outstanding feature of the greatly hyperplastic mammary glands were cysts filled with eosinophilic material which apparently exercised pressure on the lining epithelium, flattening it. Smaller cysts were surrounded by cuboid epithelium containing secretion droplets. The pituitary of the spayed partner (Rat 3) was of normal shape and colour (8 mg.). Uterus and vagina showed the signs of advanced atrophy, whereas the breast glands showed similar though less pronounced changes as seen in the intact partner.

Histologically the pituitary of the spayed partner showed the typical picture of chronic oestrogen deficiency. Numerous signet-ring cells as well as other forms of gonadotrophs were abundant in all parts of the anterior lobe. The acidophils were well granulated and of normal size, and, like the chromophobes, appeared to be reduced in numbers. A picture of the atrophic uterus as well as of a stimulated mammary gland has already been published in a previous communication to which the reader is referred (Fig. 21 and 22, Bielschowsky and Hall, 1951). The histological examination of the breast confirmed that the changes seen at autopsy were of a similar kind in both partners as far as their secretory activity was concerned.

To recapitulate, a remarkable secretory activity was found in the breast of the ovariectomised parabiont, the uterus and vagina of which showed the typical castration atrophy. In this case the stimulus responsible for "lactation" is assumed to have come from the intact partner. Oestrogenic stimulation can be excluded because of the state of pituitary, vagina and uterus of the spayed animal. At the time this observation was published we were unable to explain why such changes in the breast were seen only occasionally in spayed animals joined in parabiosis to an intact female. Revision of the material has shown that there exists a good parallelism between the presence of a pituitary tumour in the intact partner and stimulation of the breast of the ovariectomised parabiont. The drawing (Fig. 7, adapted from Gardner, 1953) illustrates the endocrine relationships which in the author's opinion are the cause of this phenomenon; the numbers indicate the sequence of events.

Rat 4. At the age of 2 months a pellet of 10 mg. consisting of 97·5 per cent cholesterol and 2·5 per cent stilboestrol was implanted into the right flank of this female. A fortnight later vaginal smears revealed the presence of cornified cells which remained the predominant cell type for 13 months. Unfortunately no smears were taken during the following 3 months, but during the week preceding death the vaginal smears were those of anoestrous. The rat was sacrificed because its weight had declined from 268 to 242 g. At autopsy a large pituitary tumour (198 mg.) was found which compressed, but had not invaded the brain. The third as well as the lateral ventricles were enlarged, the internal hydrocephalus probably being due to pressure exerted by the tumour on the base of the brain. Brownish and white areas alternated in the nodular pituitary. The ovaries were of yellowish colour, small (combined weight 53 mg.) and apparently free of corpora lutea. The uterus was of normal size (662 mg.), the breast glands

FIG. 7.—Diagram illustrating the endocrine situation in parabiotic Rats 2/3. A = intact partner. B = spayed partner. 1 = FSH. 2 = oestrogen. 3 = prolactin.

were remarkably hyperplastic and contained a fair number of milk cysts. All other organs appeared normal except the lungs which contained small areas of consolidation.

Histological investigation showed that little non-neoplastic tissue remained in the pituitary. This consisted of chromophobes and acidophils of fairly normal appearance, but the presence of basophils could not be demonstrated in the compressed rim of normal anterior lobe. The tumour itself consisted of atypical large acidophils which in some regions were nearly as numerous as non-granulated cells, whereas in others the latter predominated. Bizarre cells with giant nuclei were a common occurrence and one or more mitoses could be found in nearly all fields. Of the 3 pituitary tumours presented this neoplasm showed the greatest variation in its cellular elements (Fig. 8). In some sections, nerve fibres probably belonging to the 5th nerve were found completely surrounded by tumour cells. In all other respects especially in vascularity, the tumour resembled those described above. The absence of luteinised tissue in the ovaries was confirmed histologically (Fig. 9). The glands contained follicles in various stages of development with healthy appearing ova. Groups of pale vacuolated cells, separated by strands of spindle-shaped elements predominated in the stroma. In frozen section the pale cells were found to contain ample sudanophilic material. The uterus still showed some of the signs of oestrogenic stimulation. The mucosa was rather fibrotic and there were only a few glands present. The epithelial cells lining the uterine cavity were cylindrical or high cuboid, frequently wedge-shaped with hyperchromatic, dense nuclei and little cytoplasm. The vagina was lined by a narrow layer of squamous non-keratinising epithelium which in some areas was covered by mucified cells (Fig. 10). They stained deep red in PAS preparations. The mammary glands consisted of cystic dilated ducts with many sidebuds, often surrounded by coarse collagen fibres. The epithelium showed all the signs of secretory activity (Fig. 11).

Mucification of the vagina in absence of corpora lutea has been observed with doses of oestrogen ½ to ¼ below the minimal amounts necessary to induce oestrous (Robson and Wiesner, 1931). Since no corpora lutea were present in the ovaries, the histology of the vagina together with the vaginal smears indicate a very low oestrogen level in this animal. Whether these traces of oestrogen came still from the pellet or were secreted by the ovaries is not known. This rat was chosen out of 20 females treated in a similar manner because of the contrast between the greatly stimulated mammary glands and the minimum of stimulation of the vagina and the presence of a large pituitary tumour.

### DISCUSSION.

Like previous investigators the writer has been intrigued by the large Golgi apparatus and other morphological signs indicating secretory activity in many spontaneous or oestrogen-induced pituitary tumours. The particular circumstances prevailing in the 3 cases described make it possible to venture an opinion as to the function of their cells. Of the target organs of the adenohypophysis only the breast glands were found to be stimulated and of the known pituitary hormones only prolactin can cause secretion in the breasts of ovariectomised adult rats (Desclin, 1952).

The functional activity of the pituitary tumours of the rat is at variance with that of the acidophilic adenomata of human pathology. They secrete growth hormone whereas in rodents, gigantism has not been observed in conjunction with neoplastic changes in the adenohypophysis. What are the reasons for this discrepancy? The cytology of the normal gland provides a possible explanation. Two types of acidophils can be easily distinguished in the pituitaries of several species as for instance the rabbit (Friedgood and Dawson, 1938). Here cells with a strong affinity for carmine are found besides others which do not retain this dye. The former increase in numbers after coitus, towards the end of pregnancy and during the first days of lactation. In man, Romeis (1940) demonstrated the presence of one type of acidophils staining red, and of a second staining yellow in azan preparations, his $\alpha$ and $\epsilon$ cells. In addition he recognised a third variety of acidophils, the $\eta$ or pregnancy cells of Erdheim. In the rat the histological methods with which the writer is familiar unfortunately do not allow differential staining of two types of acidophils. The Papanicolaou-Green technique is an excellent procedure for the demonstration of acidophilic granula, even in poorly granulated cells, but all acidophils stain the same colour. General agreement has been reached as to the source of growth and of lactogenic hormones, both being secreted by acidophils (Pearse, 1952). If two types of normal acidophils exist and each is concerned with the production of one specific hormone then there is no difficulty in accepting the existence of two types of acidophilic tumours. Why in man the growth hormone producing cells undergo neoplastic changes and in rodents the prolactin secreting acidophils, I am unable to explain.

The interrelation between pituitary tumours and secretory activity of the mammary glands has been studied by Lacour (1950) in the rat. In a series of 37 oestrogen induced adenomata she found in 23 granulated as well as " chromophobic " cells with a hypertrophic Golgi apparatus. Whenever the granulated elements were present, the breast had the aspect of a " lactating " gland. She used Romeis' Kresazan technique which stained the granulated tumour cells orange. According to Lacour a few cells, having the same tinctorial qualities occur in the pituitary of the normal female rat. They become more numerous early in pregnancy and increase considerably in numbers 3 days *ante partum.* From this date onwards until weaning these orangeophil acidophils outnumber the other type. The close correlation between the presence of orangeophilic cells in the normal and tumourous pituitary and lactation changes in the breast suggested to Lacour that these elements were the source of prolactin.

The literature on experimental and spontaneous pituitary adenomata in rodents has been recently reviewed by Horning (1952) and by Gardner (1953). In contrast to the opinion of the writer, Gardner considers the oestrogen induced pituitary tumours as chromophobic adenomata, a view still shared by most authorities. Admittedly, degranulated elements are more numerous in many of these tumours than granulated forms, but can one classify all cells without granula as chromophobes? The difficulty lies in the morphological resemblance of degranulated acidophils to chromophobes. The oestrogen induced pituitary growths with functional activity cannot be chromophobic tumours because the acidophils and not the chromophobes are the source of lactogenic hormone. Another point of controversy is the nature of these pituitary growths. They are considered by some authors to be true neoplasms and conditioned growths by others, because they have been seen to regress when stimulation ceased (Nelson, 1944). The " spontaneous " tumour described in this paper did not differ histologically or functionally from the two oestrogen induced growths. This adenoma certainly was not dependant on oestrogen since it showed no sign of regression 5½ months after ovariectomy. On the other hand regression of stilboestrol induced pituitary growths after removal of implanted pellets has been observed in this laboratory.

### SUMMARY.

Three acidophilic pituitary tumours are described. One occurred in an ovariectomised rat, the second in an intact female joined in parabiosis to a spayed partner and the third in a female treated with stilboestrol, in which, however, the histology of the vagina indicated a very low oestrogen level at the end of the experiment.

The pituitary growths were found in animals having secreting mammary glands and in the case of the parabiotic pair, the breast of the spayed partner was also secreting.

The factor responsible for the stimulation of the breast in these oestrogen-deficient animals is believed to be prolactin secreted by the pituitary tumours.

### REFERENCES.

BIELSCHOWSKY, F.—(1953) *Brit. J. Cancer*, **7**, 203.

*Idem* AND HALL, W. H.— (1951) *Ibid.*, **5**, 331.

DESCLIN, L.—(1952) *Ciba Foundation Colloquia on Endocrinology*, **4**, 395.

FOLLEY, S. J.—(1952) *Ibid.* **4**, 381.

FRIEDGOOD, H. B., AND DAWSON, A. B.—(1938) *Endocrinology*, **22**, 674.

GARDNER, W. U.—(1953) *Advances in Cancer Research*, **1** 173.

HORNING, E. S.—(1952) Chapter 4, Burrows, H., and Horning, E. S., ' Oestrogens and Neoplasia.' Oxford (Blackwell).

LACOUR, F.—(1950) *C.R. Soc. Biol., Paris*, **144**, 248.

NELSON, W. O.—(1944) *Yale J. Biol. Med.*, **17**, 217.—(1952) *Ciba Foundation Colloquia on Endocrinology*, **4**, 402.

PEARSE, A. G. E.—(1952) *Ibid.* **4**, 1.

ROBSON, J. M., AND WIESNER, B. P.—(1931) *Quart. J. exp. Physiol.*, **21**, 217.

ROMEIS, B.—(1940) ' Handbuch der Mikroskopischen Anatomie des Menschen,' Vol. 6, part 3. Berlin (Springer).

### EXPLANATION OF PLATES.

FIG. 1.—Showing non-tumourous area with typical castration cells in the pituitary of Rat 1. PAS. × 400.

FIG. 2.—Area of pituitary tumour (Rat 1). A large, coarsely granulated acidophil in the centre. Note: Large Golgi apparatus in non-granulated cells. (Papanicolaou.) × 700.

FIG. 3.—Atrophic vagina (Rat 1). H. & E. × 85.

FIG. 4.—Section of mammary gland (Rat 1) having many ducts with multiple sidebuds. H. & E. × 30.

FIG. 5.—Detail of Fig. 4 showing the secretory activity of the glandular epithelium. H. & E. × 85.

FIG. 6.—Area of pituitary tumour (Rat 2). Three large acidophils with hypertrophic Golgi apparatus are seen in the centre surrounded by degranulated cells having a similar Golgi apparatus. (Papanicolaou.) × 400.

FIG. 8.—Area of pituitary tumour (Rat 4) with a dividing acidophil in the centre and atypical granulated and non-granulated cells. (Papanicolaou.) × 450.

FIG. 9.—Ovary (Rat 4.) H. & E. × 35.

FIG. 10.—Vagina (Rat 4.) H. & E. × 100.

FIG. 11.—Cystic hyperplasia of mammary gland (Rat 4.) H. & E. × 100.

Bielschowsky.

8 9 10 11

Bielschowsky.